# A five-axis parallel kinematic mirror unit for soft X-ray beamlines at MAX IV

**DOI:** 10.1107/S160057751901693X

**Published:** 2020-01-29

**Authors:** Marcus Agåker, Frieder Mueller, Brian Norsk Jensen, Karl Åhnberg, Peter Sjöblom, Jochen Deiwiks, Hans Henniger, Rainer Pärna, Jan Knudsen, Balasubramanian Thiagarajan, Conny Såthe

**Affiliations:** a Physics and Astronomy, Uppsala University, PO Box 516, SE-75120 Uppsala, Sweden; b MAX IV Laboratory, Lund University, PO Box 118, SE-22100 Lund, Sweden; c FMB Feinwerk und Messtechnik GmbH, Friedrich Woehler Strasse 2, 12489 Berlin, Germany; d Institute of Physics, Univesity of Tartu, W. Oswaldi 1, EE-51014 Tartu, Estonia

**Keywords:** beamline component, optics kinematics, soft X-ray, MAX IV

## Abstract

With the introduction of the multi-bend achromats in the new fourth-generation storage rings the emittance has decreased by an order of magnitude resulting in increased brightness. However, the higher brightness comes with smaller beam sizes and narrower radiation cones. As a consequence, the requirements on mechanical stability regarding the beamline components increases. Here an innovative five-axis parallel kinematic mirror unit for use with soft X-ray beamlines using off-axis grazing-incidence optics is presented. Using simulations and measurements from the HIPPIE beamline at the MAX IV Laboratory it is shown that it has no Eigen frequencies below 90 Hz. Its positioning accuracy is better than 25 nm linearly and 17–35 µrad angularly depending on the mirror chamber dimensions.

## Abstract 2

A five-axis parallel kinematic mirror unit, with a small form factor and high stability, for use at soft X-ray synchrotron beamlines is presented.

## 1. Introduction   

With the introduction of the new multi-bend achromat magnetic lattices, the emittance has gone from >3 nm rad for a third-generation synchrotron storage ring to <0.3 nm rad for a fourth-generation synchrotron storage ring, a reduction by an order of magnitude (Wolski, 2013 ▸; MAX IV, 2010 ▸) The drive for the reduction of the emittance has been the need to produce higher beam brightness for beamline applications. The lower emittance has not only led to an increased demand on electron beam stability (Böge, 2004 ▸; Wang *et al.*, 2008 ▸; Spataro *et al.*, 2018 ▸) but also on the beamline components, especially regarding mechanical stability and positioning accuracy. To meet this requirement, MAX IV implemented a stability policy, stating that all beamline components should be designed to work under the conditions of a vibrational level of 20–30 nm root mean square (RMS) for all frequencies >5 Hz. For components that influence resolution, spot size and beam position, such as optical components, they shall not have any natural Eigen frequencies below 55 Hz.

One way to achieve this is to make all mechanical structures compact, light and stiff. Beamline components have, however, traditionally not been designed with this philosophy in mind^1^ ^2^ ^3^, but rather with a module design that is easy to assemble and maintain. These mirror unit designs have been based on stacked systems where each movable stage is mounted on top of the previous. The positioning errors in each stage are then added to the total positioning inaccuracies as well as adding the distance between the moved object and the actual motion stage. Another way has been to use hexapods or a Stewart platform (Stewart, 1965 ▸) where six struts are attached between a common base and the positioning platform. By adjusting the lengths of the struts the platform can be positioned with six degrees of freedom. These systems tend to be quite large if they are to position the whole mirror unit including vacuum vessel and pumps, or they become quite complex with in-vacuum motors and encoders if they are mounted inside the vacuum vessel in order to reduce size.

Vacuum chambers for soft X-ray beamlines have generally been axially oriented perpendicular to the beam direction to allow access to the interior of the chamber while mounted to the beamline. This necessitates a chamber diameter in excess of the length of the mirror block to allow installation from the top. This results in large and heavy chambers that are mostly empty. In some cases, rectangular chambers, that conform more to the mirror dimensions, are used. In these cases, ConFlat (CF) flanges and standard copper gaskets cannot be used, making the vacuum seal more complicated. In both cases beam-defining apertures as well as associated vacuum equipment such as ion pumps, gauges, roughing valves *etc.* have been attached directly to the mirror chamber, adding additional weight to the moving mass.

Our new innovative mirror unit design for soft X-ray beamlines, the five-axis parallel kinematic mirror unit system, is based on a philosophy where all components have been designed to be as small as possible to reduce weight. Motion stages are positioned as close as possible to the moved object but outside vacuum, and equipment that does not need to be aligned with the mirror are separated from the mirror chamber to reduce the moved mass.

## 2. MAX IV green field   

As part of the pre-study for the establishment of the MAX IV Laboratory an extensive investigation by the Norwegian Geological Institute (NGI) was made regarding the frequency spectra present at the green field location of the new laboratory at Brunshög in the north eastern parts of Lund, Sweden. It was concluded that the vertical vibration levels at the site above 20 Hz is 4 nm RMS, while most of the maximum ground amplitudes, in the range 40–60 nm, occur at 5–20 Hz.^4^ To minimize motion of optical components the mechanical structure should not have natural frequencies similar to those present in the ambient environment. Even though most frequencies are present in the natural background, amplitudes at frequencies at the lower end of the spectrum are generally larger. If the structures have no Eigen frequencies close to those predominantly present in the frequency spectrum they will not pick up any energy from the surroundings, exciting internal vibrational modes, but rather follow the general motion of the ground. To achieve this, the mechanical structures should have minimal lever arms, weight and stacking of joints.

## 3. MAX IV mirror unit design concept   

As part of the tendering process for beamline components in the first wave of beamlines built at the MAX IV facility, a mirror unit design concept was established based on the conclusions of the stability investigations for the laboratory and experience of the staff. This design concept addresses some of the major points identified as problematic in traditional designs:

(i) No vacuum pumps should be directly attached to the mirror chamber. Any connections to the mirror chamber should be made with welded bellows, and the weight of the pump should not be borne by the mirror chamber frame.

(ii) The axis of the chamber should be along the beam path rather than perpendicular to it in order to minimize the chamber diameter and hence its size and weight.

(iii) The mirror mechanism should as far as possible be made from a single piece using machining rather than being assembled from many different parts.

(iv) The adjustment axis should be as close as possible to the mirror surface.

(v) Support points should be spread as far apart as possible within the size of the mirror chamber.

(vi) Positioning should be made out of vacuum if possible.

(vii) The mirror stand should be made of concrete/granite, and reach as close as possible to the mirror chamber.

(viii) Supports must have good, well defined, mechanical contact to the floor; three legs are a must if the system is to be well aligned, unstrained and well defined.

(ix) Mirror chamber components should be designed to work under the conditions of the MAX IV stability goal, 20–30 nm RMS for all frequencies >5 Hz. Eigen frequencies of systems that influence performance should be at least 55 Hz (‘system’ means from floor to optical component, for instance).

(x) Systems should be compact, light and stiff.

**Figure 1:**
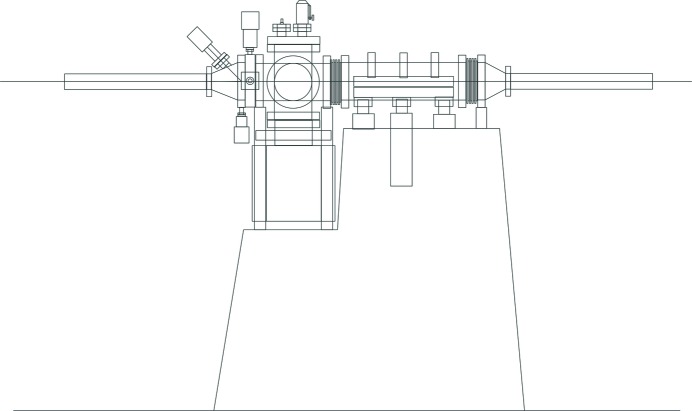
Conceptual sketch of proposed mirror unit concept.

The design concept as stated above (Fig. 1 ▸) was a common request from the Veritas, HIPPIE and Bloch (ARPES at the time) beamlines at MAX IV. Later the FinEstBeAMS beamline also joined the request.

Apart from the stability requirements the mirror units should also facilitate a pitch and yaw adjustment of ±10 mrad, 0.5 µrad per step, and roll adjustments of ±10 mrad, 5.0 µrad per step as well as a vertical and horizontal stroke of ±10 mm, 5 µm per step as stated in the optical design reports for the individual beamlines. The optical parameters of the four beamlines are, however, quite disperse, even if they are all collimated plane-grating monochromator beamlines. This required the design to be able to handle internally cooled collimating mirrors for Veritas and HIPPIE, while Bloch and FinEstBeAMS use side-cooled collimating mirrors. Different designs were also requested for the switching mirrors: an in-line sequential mounting is used for Veritas, while HIPPIE, Bloch and FinEstBeAMS are using parallel mounts. In addition, the mirror block dimensions vary between the beamline designs influencing the mirror chamber dimensions. Later beamlines like SoftiMAX have requested mirrors with dual stripes for different energy regions requiring a vertical switching. These demands require a flexible design where the individual mirror unit can be adopted to the specific optical design without unnecessarily compromising the stability requirements as defined by the design concept.

## 4. FMB Berlin mirror unit concept   

**Figure 2:**
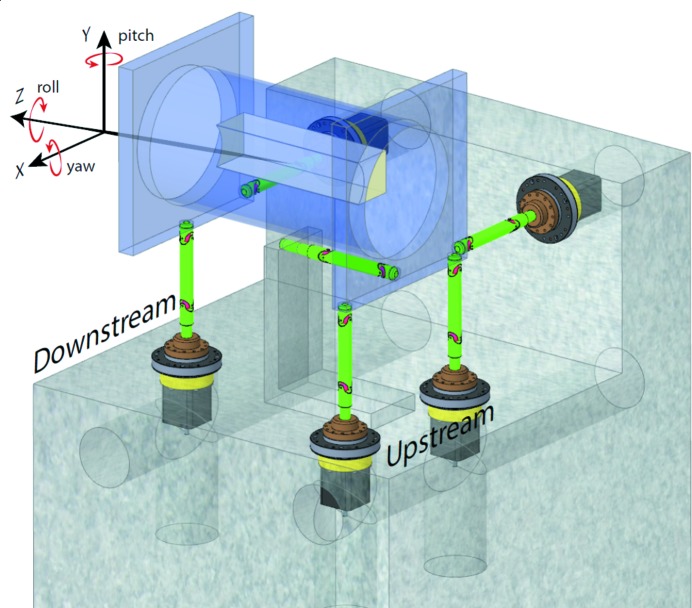
FMB mirror unit concept showing a five-axis parallel kinematic system with a small axial mirror chamber supported by five flexure legs. The mirror in this design is side deflecting, and definition of motions in the MAX IV coordinate system is shown.

Adhering as much as possible to the design proposals in the MAX IV mirror unit design concept, FMB Berlin^5^ offered a so far untested design to MAX IV, based on a five-axis parallel kinematic system, where five independently movable and one fixed flexure rods define the motion of a chamber, as seen in Fig. 2 ▸. Three legs are supporting the chamber vertically and, depending on their relative motion, allow an adjustment of height, yaw (RX) and roll (RZ). Two legs anchor the chamber horizontally and control lateral motion as well as pitch (RY). A sixth fixed leg defines the longitudinal position of the chamber. Each of the movable legs consists of a solid rod, with two gimbal flexures, attached to a linear drive, supported by a linear bearing. The fixed leg also has two gimbal flexures but does not have a drive as it does not need to be adjusted in the optical designs used at MAX IV. In this design there is an inevitable cross-talk between motions. As legs are driven along one axis of the chamber, mounting points of axes perpendicular to the motion will perform a small parasitic motion as the flexure leg holding the chamber in this direction needs to change angle to follow the driven motion. This causes the mounting point to rotate around the stationary leg drive. By moving all five legs together this can be compensated for. It is also possible to define any axis of motion or rotation within the range of the leg drives by moving all five drives at the same time.

The mirror chamber is a cylinder enclosing a monolithic mirror holder that in turn holds the mirror. The mirror unit is connected to the surrounding vacuum system by two edge-welded bellows, one at each end. Pumping is solved by using a separate dedicated vacuum chamber on its own support equipped with a 300 l ion pump.^6^

## 5. Eigen frequency   

To evaluate the design concept, a test system was built, where stability and motion accuracies could be tested. To verify the design before manufacturing, a finite-element analysis (FEA) of the CAD-model was made. Calculations were performed for two different chamber dimensions where one model has 300 mm longitudinal spacing of the legs and the other has 400 mm spacing. Calculated Eigen frequencies for the two versions are given in Table 1 ▸. The calculations were performed on CAD-models provided by FMB Berlin, simplified by removing smaller parts and changing the design of the motorized screw to make the mesh easier without changing the function. The mirror was represented by a 7 kg solid mass. Calculations were made using *SolidWorks* 7.

**Figure 3:**
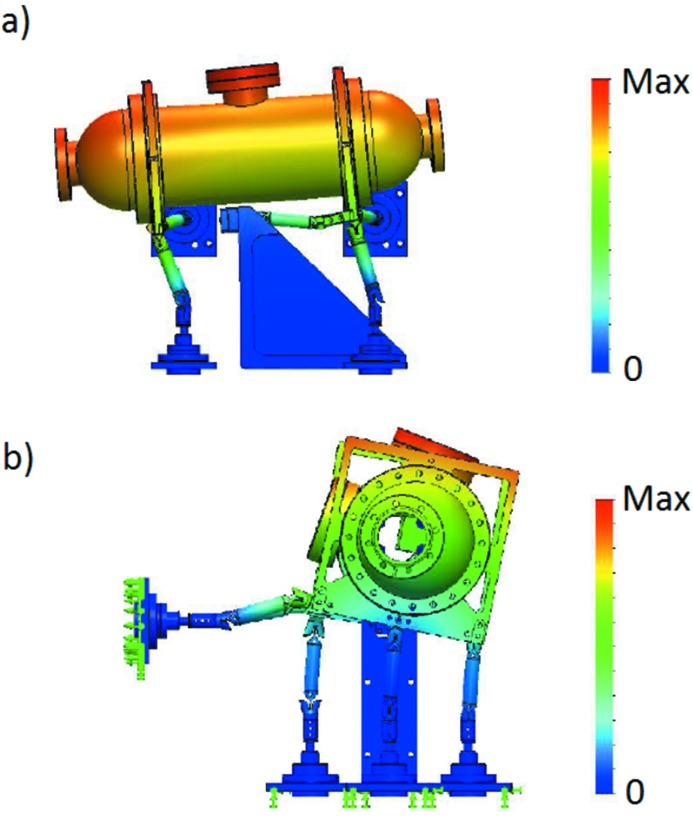
(*a*) The first Eigen mode (300 mm longitudinal leg distance) is a longitudinal rocking at 112.33 Hz. The model scale value is 0.262415. (*b*) The second Eigen mode (400 mm longitudinal leg distance) is a roll mode at 119.8 Hz. The model scale value is 0.216556. Both systems have the same types of modes but with slightly different values due to the different leg separations.

The simulations showed minimal warpage of the mirror chamber as seen in Fig. 3 ▸. Most of the deformations occur in the flexure legs at the joints which are weak by design to allow the necessary motions of the chamber. The Eigen frequencies for both systems are above 110 Hz as can be seen from Table 1 ▸. Both models show the same types of Eigen modes but the frequency for the model with 400 mm longitudinal leg distance is slightly higher due to the extra 100 mm distance between the supporting legs. Adding the granite stand (block: 925 mm × 700 mm × 500 mm; shelf: 300 mm × 175 mm × 500 mm) with a weight of ∼875 kg to the calculations shows that the frequencies are lowered by approximately 5 Hz; see Table 2 ▸.

### 5.1. Test unit   

**Figure 4:**
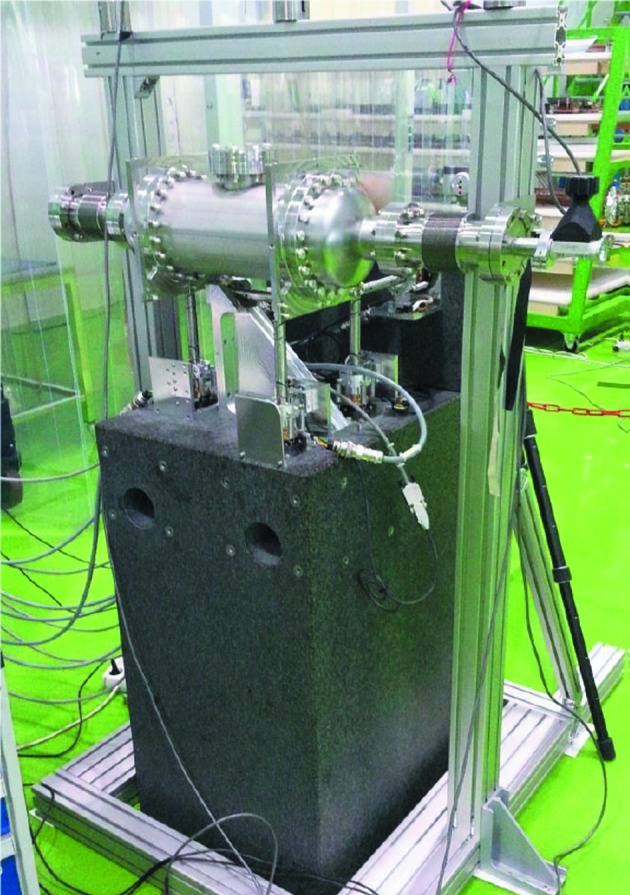
Test unit rigged for stability and motion tests at the FMB Berlin site.

Test measurements were performed on the test unit at FMB Berlin’s factory at Adlershof, Berlin, Germany. The mirror test units were attached to a granite block resting on metal washers on the workshop floor, which is coated with a few millimetres-thick layer of viscoelastic epoxy. The connecting edge-welded bellows were supported by an aluminium frame; see Fig. 4 ▸. To measure the frequency response of the mirror unit and also surrounding equipment such as the granite stand, supporting aluminium frame as well as the floor motions, four sets of Wilcoxon seismic accelerometers and a power amplifier system^8^ were used. The sensors are sensitive down to 1/20 Hz and the low-pass filter can be set between 100 Hz and 450 Hz. Amplification can be set to 10 V/*g*, 100 V/*g* or 1000 V/*g*, where *g* is the gravitational constant. For these measurements the amplifier was set to 1000 V/*g* and the low-pass filter was set at 450 Hz. The sensors weigh 670 g each which perturbs the mechanical system, downshifting the frequency response due to the added mass. One sensor was used to monitor longitudinal acceleration and two other sensors were used to either record the transverse or vertical motion. A shaker, attached to the granite block, using a force transducer, was used to put force into the system with all frequencies up to 500 Hz. The analog signals from the sensors were sampled at 1 kHz with a 16-bit USB AD-Converter from Measurement Computing^9^. Analysis was carried out using software written by B. N. Jensen in LabWIEV^10^.

**Figure 5:**
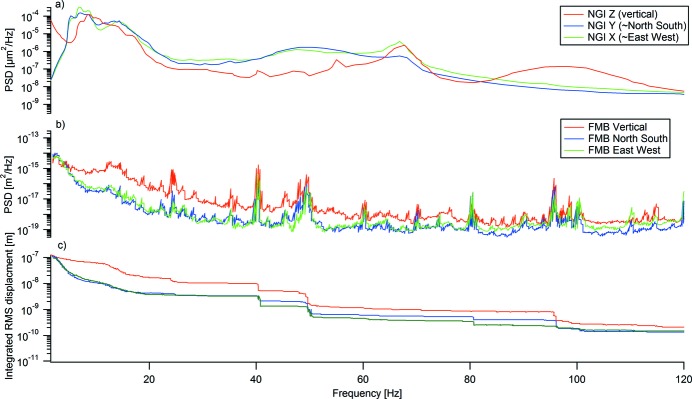
(*a*) Power spectral density (PSD) at the MAX IV green field point 1 measured by the Norwegian Geological Institute. (*b*) PSD of floor motion in the vertical, north–south and east–west directions at the prototype, FMB facility in Berlin. (*c*) Integrated RMS displacement versus high-pass cut frequency at the prototype, FMB facility in Berlin.

First the ground-level vibrations were measured at the prototype to identify external vibrational sources, seen in Fig. 5(*b*) ▸. These can be compared with point 1 of NGI’s green field measurements at the MAX IV site in Fig. 5(*a*) ▸. At the FMB site there were some stronger sources at 40, 48–49 and 120 Hz as well as some smaller ones at 60, 70 and 90 Hz as seen in Fig. 5(*b*) ▸. Vibrational levels are dominant below 15 Hz and of the order of 70–80 nm, which is larger than that measured (40–60 nm) at the MAX IV green field site as reported in the NGI report. During measurements it was evident that the surrounding vibrational levels and spectrum changed due to the activities at the factory but it is not considered to have affected the analysis of the measurements.

**Figure 6:**
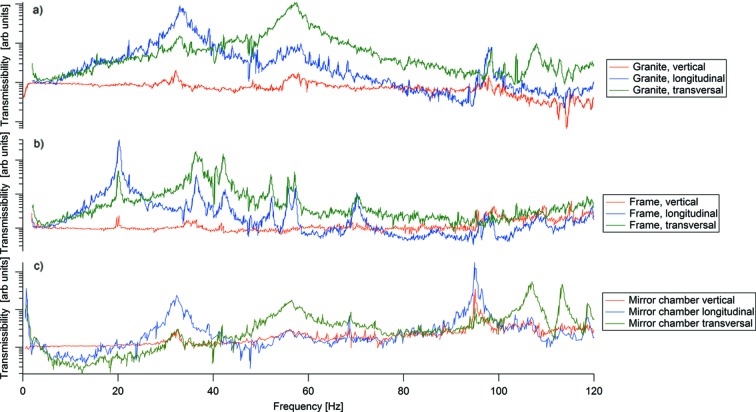
Vertical, longitudinal and transversal transmissibility of (*a*) the granite block relative to the floor, (*b*) the aluminium frame relative to the floor and (*c*) the mirror chamber relative to the floor.

To rule out contributions from the granite support and the frame holding the edge-welded bellows, transmissibility measurements relative to the floor were made for the granite block and frame, respectively, as seen in panels (*a*) and (*b*) of Fig. 6 ▸. Generally, an Eigen mode should be visible in multiple channels as there is cross-talk between different directions and the placements of the accelerometers are never completely aligned with one single mode of motion. Furthermore, it is expected that the first Eigen mode in each direction should be the strongest one in that channel. By identifying resonances seen in several of the channels for the granite and aluminium frame, Eigen modes were identified at 33, 57 and 98 Hz for the granite block and at 36, 42, 52, 57 and 70 Hz for the aluminium frame. In panel (*c*) of Fig. 6 ▸ the transmissibility of the mirror chamber relative to the floor is shown. Considering the identified resonances in the granite block and the supporting aluminium frame, and considering that the first Eigen mode should be the strongest resonance, it is clear that the first resonance that can be connected to only the mirror unit is at 95 Hz. This seems to be connected to both a longitudinal as well as a vertical motion. The first mode in the FEA, 112 Hz as seen in Fig. 3(*a*) ▸, is a rocking mode around the transvers axis which would include some vertical as well as longitudinal motion. Several other modes in the 100–120 Hz range regime can also be seen in the measurements. The addition of the motion sensors to the system will have lowered the apparent frequencies due to their masses and, considering this, the measured results are well in agreement with the FEA calculations.

Perturbing the system by vacuum forces/no vacuum forces or positioning the system to its extremes has very little influence on the Eigen frequencies. There is also no cross-talk from the surrounding environment through the edge-welded bellows. During these measurements the granite block was not grouted to the floor which would influence the results.

### 5.2. HIPPIE M4   

**Figure 7:**
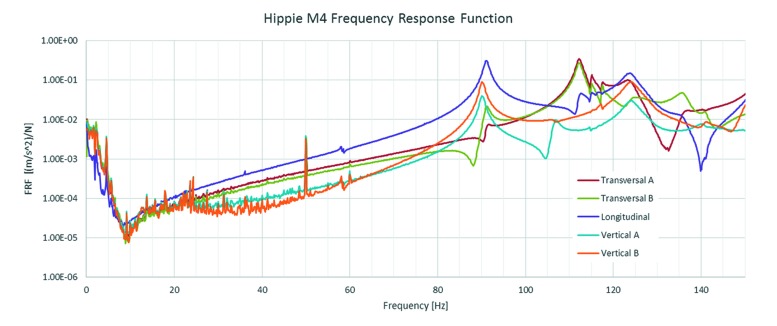
Frequency response function in vibrations for the HIPPIE M4 mirror chamber.

After installation, alignment and integration into the beamline control system, a new study was made of the vibrational stability of the final design mirror unit at HIPPIE M4 (refocusing mirror prior to the experimental endstation) using the same seismic accelerometer equipment used at FMB Berlin, see Section 5.1. The lowest Eigen mode found is around 90 Hz, some 20 Hz lower than the FEA calculations as seen in Fig. 7 ▸ and 5 Hz lower than measurements on the test unit. The shift in frequency is most likely coupled to changes in the final mechanical design as well as the instalment at the beamline, including the real mirror and cabling.

## 6. Positioning   

To verify the positon accuracy and long-term stability of the system a series of measurements were performed both on the test unit at FMB Berlin and at the installed M4 (refocusing mirror) at Veritas B.

### 6.1. Test unit   

**Figure 8:**
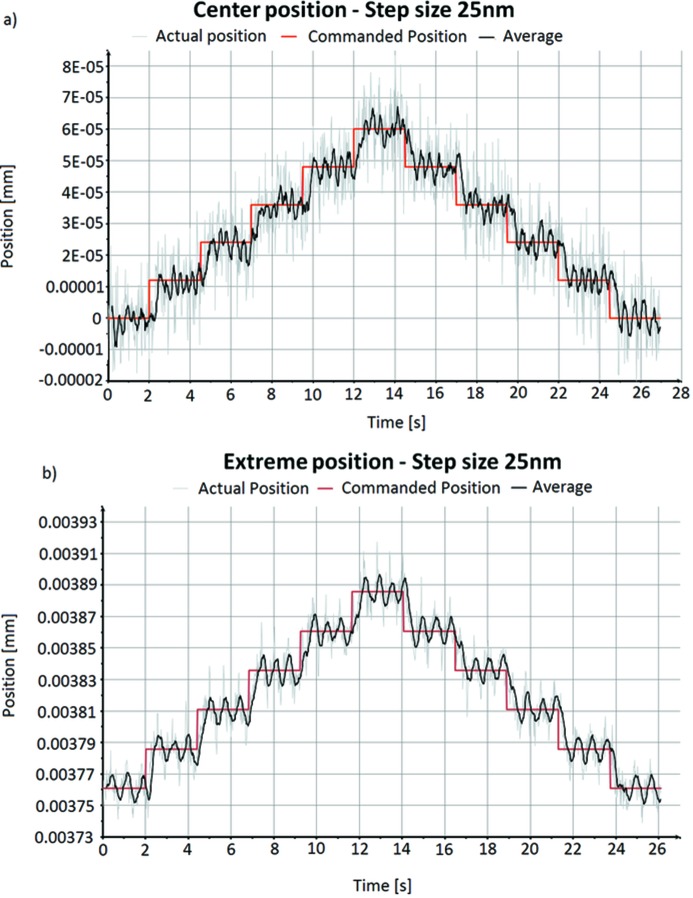
(*a*) Positioning of the mirror chamber with 12.5 nm steps at the centre position. (*b*) Positioning of the mirror chamber with 25 nm steps at the limit of the leg motion range.

Motion tests were made on the test unit using a laser interferometer^11^, monitoring the chamber motion as a function of leg movements. To reposition a mirror, all five legs are driven simultaneously even though only certain combinations are required to execute individual motions. This is to compensate for the inevitable cross-talk between the different legs when the chamber is moved. In these experiments the chamber was moved stepwise by increments of 12.5 nm and 25 nm in first one direction then in the reverse direction. Measurements were made with the leg drives at the centre position as well as at extreme positions close to the mechanical limits. During these motions the leg drives were operated in a closed loop by the encoders of each leg drive. Results are presented in Fig. 8 ▸. As can be seen, a distinct staircase pattern, representing the individual positioning steps, can be seen. The mirror unit returns to the starting positon after the positioning cycle without backlash. The motion of the chamber follows the leg drive steps with a high accuracy even when the legs are at high deflections. The small periodic oscillations in the measurements are most likely from the measurement setup.

The angular positioning accuracy of the mirror chamber is 25 nm per full step resulting in ∼17 µrad per step angular resolution for pitch and yaw, which depends on the chamber length and the resulting longitudinal distance between the linear acting legs. For roll the transverse distance between the legs sets the angular step size to ∼35 µrad per step due to the shorter distance between the legs. Using microstepping of the motors, 0.049 nm per step can be reached at 512 microsteps. The maximum stroke for pitch and yaw of the mirror chamber is ±33 mrad; for roll it is limited to ±10 mrad (±45 mrad without bellows) by the small allowed torque of welded bellows connecting the mirror chamber to the surrounding vacuum system. The motion range also depends on the chamber size and resulting distance between the legs. The smaller the chamber, the larger the angular range but the coarser the resolution.

### 6.2. Veritas M4   

In operation at MAX IV, the motors are kept in a closed-loop operation relying on optical linear absolute encoders from Renishaw^12^ mounted at the drive of the flexure legs. The closed-loop deadband is set to ten half-steps (∼125 nm drift). The encoders use a 32-bit BiSS-C protocol with 1 nm resolution. The motors are driven with a IcePAP motor controller, a collaboration between MAX IV, ESRF and ALBA (Janvier *et al.*, 2013 ▸). Motion tests performed on the Veritas B M4 (the last mirror unit before the endstation on the Veritas B branch) mirror unit verify the positioning accuracy and stability of the system as measured at FMB, here presented by vertical position and yaw angle. Veritas B M4 is not grouted to the floor but rests on three support screws and it is attached to the beamline vacuum system, which might influence the results. In these measurements the Renishaw absolute encoder is sampled, measuring the mirror chamber motion indirectly, in contrast to the laser interferometer measurements at FMB which measured the chamber motion directly.

**Figure 9:**
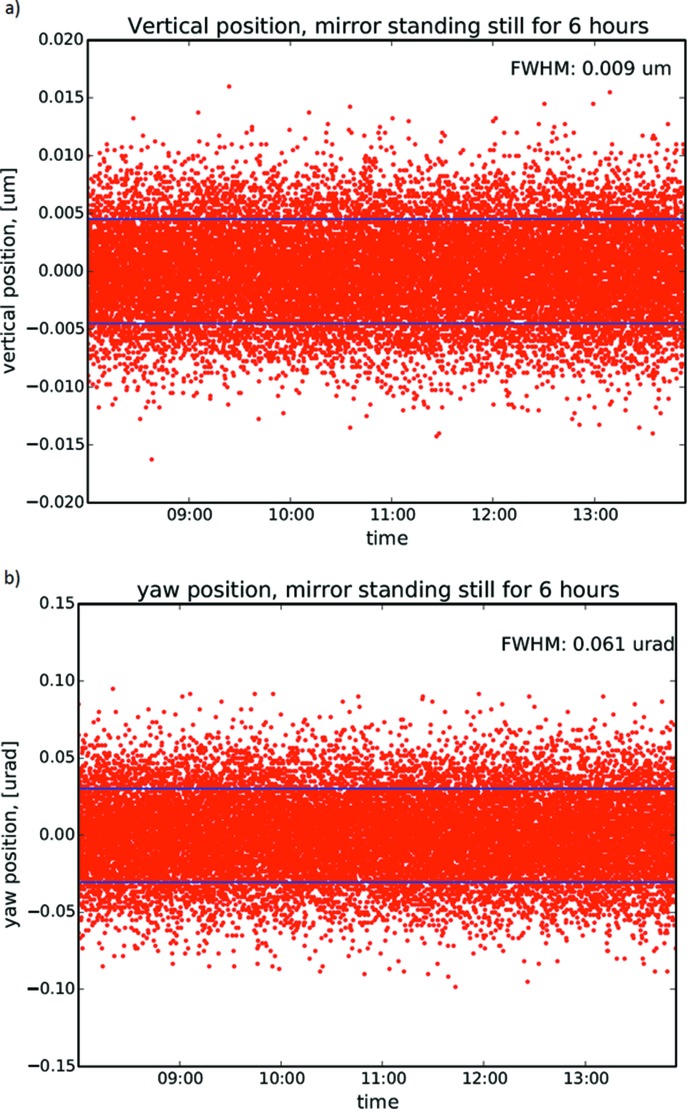
The mirror leg drives are in closed-loop operation but no request for motion is issued. The position is held for 6 h while the absolute position encoders are sampled every second. (*a*) The vertical position is shown. The FWHM of the noise is 0.009 µm which is within the deadband of the closed loop showing the long-term position stability of the system. (*b*) The yaw angle is shown. The FWHM of the noise is 0.061 µrad which is within the deadband of the closed loop, showing the long-term angular stability of the system.

**Figure 10:**
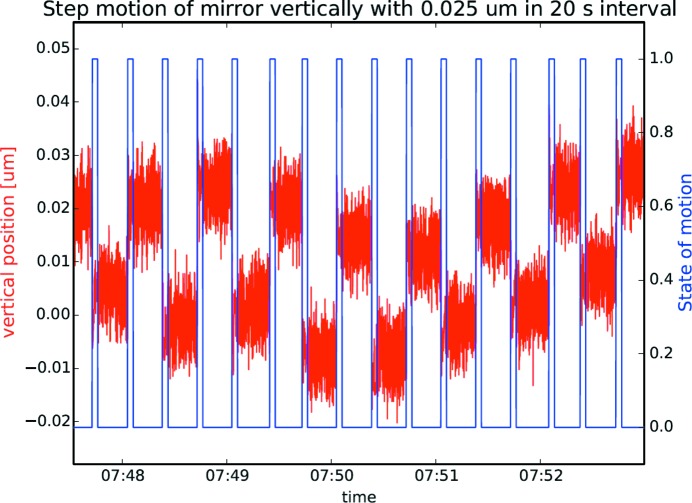
The vertical position is altered every 20 s by stepping the motors with two half-steps (0.025 µm) while the absolute Renishaw encoder is sampled. Overlaid is also the state of the motion where zero indicates that the motion has come to a stop and a measurement could start. The 0.025 µm step is well resolved but a variation in absolute position is present. Motions are within the deadband of the closed loop so no corrections are activated in this case.

In Fig. 9(*a*) ▸ it can be seen that the average full width at half-maximum (FWHM) fluctuations of the vertical mirror position during a 6 h measuring period is 9 nm. In Fig. 9(*b*) ▸ a similar measurement, monitoring the yaw motion (rotation around the mirror normal, see Fig. 2 ▸), is shown. The average over a 6 h period is 61 nrad FWHM. In both cases the long-term mechanical stability is within the deadband of the closed loop, meaning that no position correction is issued by the system. Fig. 10 ▸ shows the result of stepping the leg drives vertically, the *x*-direction in Fig. 2 ▸, with two half-steps (25 nm) back and forth every 20 s. Each step is clearly visible but there is a drift in the absolute positioning between steps. This might be due to mechanical backlash and heating in the system. The drift is smaller than the deadband of the closed loop so no active correction takes place during the cycling.

### 6.3. Conclusions of tests   

As can be seen from the tests at FMB Berlin, HIPPIE and Veritas, the mirror units fulfil the design goal for stability and position accuracy as requested by MAX IV Laboratory.

## 7. Final designs   

**Figure 11:**
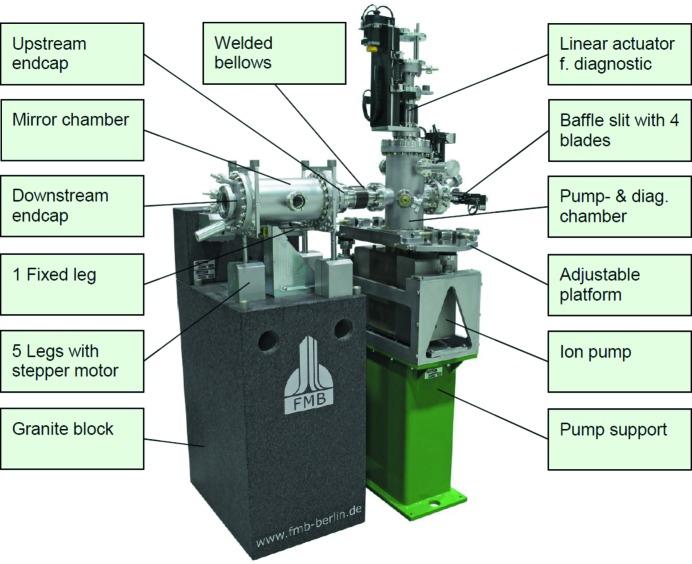
Final design of the MAX IV mirror units with included components.

The final design of the MAX IV mirror units consists of the mirror chamber mounted on a supporting granite block. Upstream from the mirror chamber is a second chamber that holds the vacuum pump, a diagnostic tool and a beam-defining aperture. This chamber is supported by its own stand and is completely decoupled from the mirror unit by edge-welded bellows as seen in Fig. 11 ▸.

The granite block holding the mirror units consists of two pieces, glued and bolted together. One large rectangular block goes from 30 mm above floor level to the bottom of the vertical leg drives, and a smaller shelf is attached to the top of the larger block to hold the horizontal leg drives. Seats for the leg drives are machined directly into the granite blocks, allowing a stable mounting of the legs to the reference surfaces. The granite blocks are then under-grouted to the experimental hall floor, essentially moving the floor level up to the top of the granite blocks and the base of the flexure legs, minimizing the distance from the floor reference to the mirror surface.

The stand for the pumping and diagnostic chamber is made of steel and aluminium as the stability requirements for these components are not as strict as for the mirror chamber.

**Figure 12:**
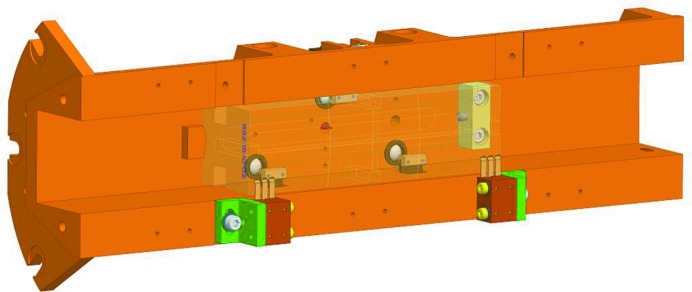
Monolithic aluminium holder showing the ceramic support balls, surface electrodes and a ghosted-out mirror block.

Inside the mirror chamber the mirror is mounted in a monolithic aluminium holder (Fig. 12 ▸) that is fixed to the chamber at one end axially and radially mounted with spring washers at the other end. The mirror holders are equipped with an isolated electrode, mounted above the mirror surface to measure the electron yield from the mirror, providing a means to measure the flux at each mirror even if they are grounded (as for the water-cooled mirrors). Each mirror is clamped to the holder by spring-loaded clamps pressing the mirror block onto ceramic balls, defining the mirrors position relative to the holder. Where full balls cannot be used a thin Kapton film is used together with spherical metal studs. The mirror holders are also equipped with two thermocouples mounted at the rear side of the mirror block, one at the centre and one at the upstream end of the mirror. For the collimating mirrors an uncooled copper block protects the mirror short end from accidentally being hit by the synchrotron beam during alignment. One of the thermocouples can be alternatively mounted to this block. For the uncooled mirrors there are, in addition to the electrode, also two contacts for drain current measurements directly from the mirror surface for the same purpose as the electrode.

The compact design of the mirror chamber and the internal mirror holder has further benefits. The small mirror chamber containing the optics and holder can easily be dismounted from the five flexure legs without affecting the internal mirror mounting. The vacuum chamber can then be brought to a clean environment for installation and service of the mirrors. As the mirror blocks are installed in their holders before being inserted into the vacuum chamber, all connections can be checked while having full access to the mirror in its mount. This also allows for metrology of the mirror surface while mounted without interference from the vacuum vessel.

**Figure 13:**
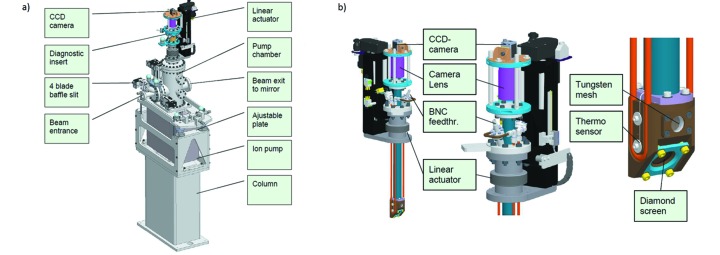
(*a*) Pumping unit exterior, (*b*) diagnostic tool.

The pumping unit, as seen in Fig. 13(*a*) ▸, mounted upstream of the mirror units has a 300 l Gamma Vacuum ion pump as well as an angular roughing valve. The system can reach 5 × 10^−10^ mbar ultimate pressure. The conductance between the mirror chamber and the pumping chamber is determined by the length and diameter of the connection bellows which defines the pressure in the mirror chamber. The pumping unit also has a four-blade beam-defining aperture^13^. The blades have a stroke of 20 to 32 mm (depending on the beam size) and can be positioned with a 1 µm accuracy. The stroke of the blades allows for some overlap to enable the aperture to be moved off-centre. Each blade is isolated and the drain current from each blade can be measured individually. There is also a diagnostic tool, see Fig. 13(*b*) ▸, with a fluorescent screen^14^, gold mesh and a diode^15^. The fluorescent screen (YAG) has an etched pattern with a 2/10/20 mm pitch to allow for beam size measurements. The screen is mounted at a 60° incidence to the beam and viewed by an outside vacuum camera from above. The gold mesh has a 100 lines inch^−1^ grid pattern with 85% transmission. The diode has a 10 mm × 10 mm active surface. The diagnostic tool is motorized and monitored with an absolute Renishaw encoder to allow it to be positioned repeatedly at the same position.

The mirror units are equipped with target nests for use with a laser tracker^16^. These are used with a global coordinate system at the laboratory to pre-position the mirror unit before final alignment with light. With this pre-alignment only minor motions of the mirror units are needed.

The mirror unit design is individualized for each mirror according to the optical design of the beamlines. This includes different mirror block sizes, extra-short exit arms, dual or sequential mounts as well as internally cooled and side-cooled mirrors. To facilitate the individual requirements some key parameters, like chamber diameter or length, can be changed including individualized endcaps. The basic concept of the units is, however, always kept the same. The main parameters of the mirror units delivered are shown in Table 3 ▸.

The final designs of the mirror unit can handle chambers ranging in diameter from 150 to 200 mm and lengths between 200 and 400 mm depending on mirror block sizes. Mirror blocks range from 94 mm to 550 mm in length with cross sections varying from 40 mm × 40 mm, 60 mm × 60 mm and 80 mm × 40 mm with single and dual mounts (see Table 3 ▸).

### 7.1. Known issues   

During initial commissioning of the beamlines it has been noted that there is a drift in the pointing of the beam as a function of time and power load from the synchrotron. Investigations have been made into the origin of this phenomena. First the heating of the mirror block itself causes the beam to drift before thermal stabilization occurs. This is not unexpected. Temperature measurements on the first mirror unit have also shown an asymmetric heating of the chamber and the flexure legs, causing a second and third stabilization time.

It has also been noted that the opening in the front-end allows a larger part of the off-axis radiation cone [see ch. 5.4.2 of Attwood (1999 ▸)] into the first mirror unit than necessary. This might allow higher-order more energetic photons, second and fourth harmonics, to enter into the mirror unit with larger angular divergence and that this might be the cause of the asymmetric heating issues of the mirror chamber and the flexure legs. At Veritas a reduction of the front-end aperture to only allow on-axis light into the mirror chamber reduced the drifting problems significantly. For HIPPIE a closed-loop positioning software routine was introduced to reposition the mirror pointing to correct for the thermal drifts. At Bloch it was noted that after a 2.5 h thermalization period the drifting stopped and the pointing stayed stable for the duration of operation. At FinEstBeAMS no significant beam drifts were observed during operation; however, beamline requirements for beam size is modest compared with Veritas, HIPPIE and Bloch. We do not assign these heating issues as a direct result of the design but rather as a combination of a higher than expected radiation load combined with a compact design. To combat the heating, extra cooling of the chambers might be implemented in the future as well as extra radiation protection. Apart from these heating issues with the first mirror in the beamline, all other mirror units perform as expected.

## 8. Conclusion   

At MAX IV Laboratory an innovative five-axis parallel kinematic mirror unit (FMB patents concerning the design: EP 3198321; US 9846294; JP 621813; CN ZL 2014800819770.9; CA 2957870) for use with soft X-ray beamlines has been developed together with FMB Berlin. It has five degrees of freedom of motion, lateral and vertical translation as well as yaw, roll and pitch adjustment. Translations can be made with 25 nm steps over a range of 10 mm, monitored with absolute linear encoders. Angular motions can be made with 0.5 µrad steps over a range of more than 20 mrad. The mirror positioning system is fully decoupled from diagnostic, beam-defining aperture and vacuum equipment, minimizing the movable mass in the system. Due to its light weight and compact design the achieved lowest Eigen frequencies are above 90 Hz, surpassing the stability goals at MAX IV with a wide margin. These mirror units are now used at Veritas, HIPPIE, Bloch, FinEstBeaMS, SoftiMAX, FlexPES, MAXPEEM and Nanomax (leg drives but not the chamber and mirror holders).

## Figures and Tables

**Table 1:** Eigen frequencies for longitudinal distances between the legs of 400 mm and 300 mm Granite stands are not included in these values.

400 mm longitudinal leg distance		300 mm longitudinal leg distance	
Mode number	Frequency (Hz)	Mode number	Frequency (Hz)
1	115.26	1	112.39
2	119.82	2	123.53
3	207.39	3	191.75
4	212.98	4	215.63
5	269.87	5	272.02

**Table 2:** Eigen modes of model with 400 mm longitudinal leg distance, including the granite stand

Mode number	Frequency (Hz)
1	109.56
2	114.07
3	197.81
4	206.45
5	215.8

**Table:** 

Common parameters			Range				Full-step resolution			
Reflection angle (horizontal/side-bouncing)			≥175°							
Lateral translation (lateral leg drives)			±5 mm				0.025 µm			
Height translation (vertical leg drives)			±5 mm				0.025 µm			

**Table:** 

Variable parameters	Long version			Standard version		Thick version			Short version	
Nominal chamber diameter (mm)	150			150		200			150	
Mirror bulk length, (*L*) (mm)	380 ≤ *L* ≤ 550			94 ≤ *L* ≤ 360		94 ≤ *L* ≤ 360			94 ≤ *L* ≤ 250	
Mirror bulk cross section (W × T) (mm)	40 × 60			40 × 40		2 × 60 × 60			40 × 40	
	60 × 60			40 × 60		2 × 80 × 40			40 × 60	
				80 × 40					80 × 40	
Longitudinal leg distance (mm)	400			300		300			220	
Transversal leg distance (mm)	220			220		270			220	

**Table:** 

Resulting DOF (separately moved)	Stroke (mrad)	Resolution (µrad)		Stroke (mrad)	Resolution (µrad)	Stroke (mrad)		Resolution (µrad)	Stroke (mrad)	Resolution (µrad)
Yaw (RX)	±25	0.13		±33	0.17	±33		0.17	±45	0.23
Pitch (RY)	±25	0.13		±33	0.17	±33		0.17	±45	0.23
Roll (RZ)†	±45	0.23		±45	0.23	±37		0.19	±45	0.23

† The roll rotation is limited to ±10 mrad by the flexibility of the welded bellows (diameter, length and the number of membrane pairs).

## Footnotes

1
https://www.bestec-berlin.de/category/optics/product-category/mirror-unit/.

2
https://www.irelec-alcen.com/en/synchrotron-beamline-optical-systems/complete-mirror-systems.

3
http://www.toyama-en.com/synchrotron/mirror_chamber_kb.html.

4 Report by NGI on MAX IV site ground vibrations, Askelerator i Lund – vibrasjonsmåling og vurdering av grenseverdier, 20091528-00-5-R, 19 February 2010, rev 1.

5 FMB Berlin, https://www.fmb-berlin.de/.

6 Gamma Vacuum Ion pump, https://www.gammavacuum.com/index.php/product?id=16.

7 Solidworks Simulation, https://www.solidworks.com/product/solidworks-simulation.

8 Wilcoxon Seismic Accelerometers, 731A/P31 seismic accelerometer and power amplifier system.

9 16-bit USB AD-Coverter from Measurement Computing, type USB-1616FS with collecting software *TracerDAQ Pro* V.2.1.6.1.

10 National Instruments Labwiev programing software, https://www.ni.com/sv-se/shop/labview.html.

11 Renishaw XL 80 (quadrature), https://www.renishaw.com/en/xl-80-laser-system--8268.

12 Renishaw RESOLUTE RL32BAT001B05F, Renishaw plc, Data sheet L-9517-9448-04-H, June 2018.

13 Uncooled aperture S3020, https://www.fmb-oxford.com/products/beamline-components/slits/in-flange-slits/.

14 YAG-Ce by Crytur

15 AXUV100G, Opto Diode Corp.

16 Leica 401 from Hexagon, https://www.hexagonmi.com/solutions/case-studies/energy-and-power/leica-absolute-tracker-at401-with-spatialanalyzer.

## References

[bb8] Attwood, D. (1999). *Soft X-rays and Extreme Ultraviolet Radiation. * Cambridge University Press.

[bb1] Böge, M. (2004). *Proceedings of the 9th Particle Accelerator Conference (EPAC2004)*, 5–7 July 2004, Lucerne, Switzerland, pp. 211–215.

[bb2] Janvier, N., Clement, J. M., Farjado, P. & Cuni, G. (2013). *Proceedings of the 14th International Conference on Accelerator and Large Experimental Physics Control Systems (ICALEPCS2013)*, 6–11 October 2013, San Francisco, CA, USA, pp. 766–769. TUPPC081.

[bb7] MAX IV (2010). *MAX IV Detailed Design Report*, https://www.maxiv.lu.se/accelerators-beamlines/accelerators/accelerator-documentation/max-iv-ddr/

[bb3] Spataro, C., Lincoln, F. & Sharma, S. (2018). *Proceedings*, 10th Mechanical Engineering Design of Synchrotron Radiation Equipment and Instrumentation (MEDSI2018), 25–29 June 2018, Paris, France, pp. 267–269. WEPH29.

[bb4] Stewart, D. (1965). *Proc. Inst. Mech. Eng.* **180**, 371–386.

[bb5] Wang, X., Chen, L., Yan, Z., Du, H. & Yin, L. (2008). *J. Synchrotron Rad.* **15**, 385–391. 10.1107/S090904950800650X 18552432

[bb6] Wolski, A. (2013). *Proceedings of the CAS-CERN Accelerator School: Advanced Accelerator Physics*, 19–29 August 2013, Trondheim, Norway, edited by W. Herr. CERN-2014–009. Geneva: CERN.

